# Potential Aspects of the Use of Cytokines in Atopic Dermatitis

**DOI:** 10.3390/biomedicines12040867

**Published:** 2024-04-15

**Authors:** Magdalena Krupka-Olek, Andrzej Bożek, David Aebisher, Dorota Bartusik-Aebisher, Grzegorz Cieślar, Aleksandra Kawczyk-Krupka

**Affiliations:** 1 Clinical Department of Internal Diseases and Geriatrics, Chair of Internal Diseases, Dermatology and Allergology in Zabrze, Medical University of Silesia, 40-055 Katowice, Polandandrzej.bozek@sum.edu.pl (A.B.); 2 Doctoral School, Medical University of Silesia, 40-055 Katowice, Poland; 3 Department of Photomedicine and Physical Chemistry, Medical College of the University of Rzeszów, 35-959 Rzeszów, Poland; daebisher@ur.edu.pl; 4 Department of Biochemistry and General Chemistry, Medical College of the University of Rzeszów, 35-959 Rzeszów, Poland; dbartusikaebisher@ur.edu.pl; 5 Department of Internal Diseases, Angiology and Physical Medicine, Centre for Laser Diagnostics and Therapy, Medical University of Silesia in Katowice, Batorego 15, 41-902 Bytom, Poland; cieslar1@tlen.pl

**Keywords:** cytokines, atopic dermatitis, treatment

## Abstract

Atopic dermatitis (AD) is an abnormal inflammatory response in the skin to food, environmental IgE, or non-IgE allergens. This disease belongs to a group of inflammatory diseases that affect both children and adults. In highly developed countries, AD is diagnosed twice as often in children than in adults, which may possibly be connected to increased urbanization. The immune system’s pathomechanisms of AD involve humoral mechanisms with IgE, cellular T lymphocytes, dendritic cells occurring in the dermis, Langerhans cells occurring in the epidermis, and other cells infiltrating the site of inflammation (eosinophils, macrophages, mast cells, neutrophils, and basophils). Cytokines are small proteins that affect the interaction and communication between cells. This review characterizes cytokines and potential aspects of the treatment of atopic dermatitis, as well as new strategies that are currently being developed, including targeting cytokines and their receptors.

## 1. Atopic Dermatitis

Atopic dermatitis (AD) is an abnormal inflammatory response in the skin to food, the environmental IgE, or IgE-independent allergens that is characterized by chronic inflammation, irritation, and redness of the skin. Inflammation in the skin is caused by the following factors:

- T-helper type 2 Th2-type cytokines (IL-4, IL-5 and IL-13) [1];
- Macrophages [1];
- Eosinophils [1];
- Basophils [1];
- Invariant natural killer T cells (iNKTs) [1];
- Innate helper type 2 cells (ILC2) [1].

At present, the pathological processes of AD are not fully understood. Atopic dermatitis is believed to be a consequence of genetic vulnerability, dysfunction of the immune system, environmental factors, and dysfunction of the epidermal barrier [2].

Atopic dermatitis is a chronic disease that usually begins in infancy or childhood and is phenotypically divided into extrinsic and intrinsic forms [2].

The more common form of atopic dermatitis is characterized by high levels of total serum IgE and the presence of specific IgE for environmental and food allergens.

The intrinsic (non-allergic) form of atopic dermatitis occurs less frequently (20%), more often in women, and is characterized by a normal level of total IgE and the absence of specific IgE.

Historically, the above terminology has its roots in descriptions of asthma reported by Rackeman in 1947. In the late 1980s, this terminology was also adopted for AD. According to the nomenclature of the EAACI Nomenclature Task Force, the term “atopic” can be used to describe various subtypes of atopic dermatitis. However, the term “internal” refers to non-allergic AEDS, in which there is a normal IgE level, no accompanying respiratory diseases, and negative skin tests for inhaled or food allergens.

Therefore, total IgE can be considered an indicator that allows for external dermatitis to be distinguished from internal atopic dermatitis in both children and adults.

Intrinsic atopic dermatitis is characterized by a normal skin barrier, lower expression of IL-4, IL-5, and IL-13, and higher expression of IFN-gamma. The development of extrinsic atopic dermatitis is inhibited by Th1.

Clinically, this form is characterized by the Dennie–Morgan infraorbital fold, relatively late onset of the disease, absence of eczema on the hands and feet, absence of ichthyosis vulgaris, absence of palmar hyperlinearity, lower Staphylococcus aureus colonization, and a milder disease course [3].

## 2. Epidemiology

Atopic dermatitis is also known as atopic eczema or neurodermatitis. This disease belongs to the group of inflammatory diseases that affect both children and adults and is connected with increased urbanization. The general prevalence of is 15–20% among children and over 10% among adults [4]. The incidence of this disease has increased 2–3 times over the last decades in industrialized countries. According to the reports of the International Study of Asthma and Allergy in Childhood (ISAAC), the incidence of this disease varies greatly around the world and, at the age of 6–7 years, ranges from 0.9% in India to 22.5% in Ecuador, with the highest values in Asia and Latin America. For older children aged 13–14, it has been shown that the prevalence ranges from 0.2% to 24.6%. The highest prevalence is found in Great Britain and New Zealand, and a prevalence above 15% has been reported in Finland, Africa, Latin America, and Oceania. The annual incidence of AD in adults is dependent on the region, and was 4.4% in Europe, 4.9% in the USA, and 3.5% in Canada. Each year, AD is diagnosed with an incidence ranging from 1.2% (Asia) to 17.1% (EU), and the prevalence of symptoms ranges from 3.0% to 17.7% [5].

In highly developed countries, AD is diagnosed twice as often in children as in adults [6,7]. For example, in Great Britain, AD affects, on average, 11–20% of pediatric patients and about 5–10% of adults [8]. In the United States, AD affects 7% of adults and 13% of children [9].

Atopic dermatitis is characterized by intense itching and recurrent eczema with a heterogeneous appearance and clinical course [10,11].

## 3. Etiopathogenesis

In recent few years, we have made intensive progress in understanding the epidemiology, pathophysiology, genetics, and treatment of AD.

Abnormal interactions between the immune system, nervous system, and skin cells play a key role in the pathophysiology of atopic dermatitis. The interactions involve Toll-like receptors (TLRs), neurotransmitters, cytokines, their associated receptors, and molecules involved in signal transduction. Abnormal interactions in the aforementioned systems are responsible for the main symptoms, i.e., itching and pain.

The mechanisms associated with the persistence of itching and pain in patients with atopic dermatitis can be divided into cellular and molecular mechanisms. Different types of cells are responsible for itching and pain. These include Schwann cells, astrocytes, or oligodendrocytes that communicate with immune cells, mediating chronic itching and pain. In turn, in neuropathic pain, the basic role is played by glial cells, which are activated after nerve damage and are responsible for the persistence of neuropathic pain. Pain signals activate the synthesis of adenosine triphosphate (ATP) and matrix metalloproteinases 9 (MMP-9), nerve growth factor (NGF), and ATP-activating DRG neurons, causing peripheral sensitization. In turn, astrocytes are activated in the central nervous system and produce pro-inflammatory CXCL1 and CCL2 molecules, causing pain. All these factors activate spinal cord microglia, which also leads to the release of pro-inflammatory cytokines acting on pain dorsal horn neurons.

Other cells responsible for itching and pain in atopic dermatitis include keratinocytes, which secrete TSLP, and endothelin-1 (ET-1) and NGF, which are factors that activate nerves and lead to increased sensitivity and dysfunction of the skin barrier.

Other cells involved in this process are mast cells (acting through the neurokinin receptor and the Mas-related G-protein-coupled receptor (Mrgpr)X2), macrophages and basophils producing leukotriene C4 (which activates sensory neurons and leads to severe itching through TRPV1 and TRPA1, and the cysteinyl leukotriene receptor 2 (CysLTR2)) [12].

The main pathway leading to AD is the TH2 immune response. A characteristic phenomenon of AD is an imbalance between TH1 and TH2 lymphocytes.

The balance between TH1 and TH2 lymphocytes determines the correct immune response [13].

At the beginning of the disease, there is a predominance of TH2 lymphocytes secreting interleukins (IL): IL-4, IL-5, IL-6, IL-10, and IL-13. This period is called the early phase of the IgE-dependent immune response. The increase in IL-4 and IL-13 stimulates B cells to produce IgE. Moreover, the production of IL-4 and IL-13 is increased while the production of interferon γ (IFN-γ) and tumor necrosis factor α (TNF-α) is reduced; in this phase, conditions occur that increase the susceptibility of the skin to Staphyloccocus aureus infections. Staphylococcus aureus strains produce exotoxins with superantigen properties, contributing to the strengthening of the skin immune response. In the chronic phase (the so-called late phase of the IgE-dependent immune response), there is a predominance of TH1 lymphocytes, which produce IFN-γ, TNF-α, IL-2, and a number of other pro-inflammatory cytokines responsible for chronic inflammatory lesions of eczema [10,11].

The genetics of AD are not fully understood. Research suggests that several genes may be involved in the development of the disease, with one of them being the CARD11 gene. CARD11 is an important mediator of antigen receptor signaling in lymphocytes. Loh et al. showed experimentally that the DOK3 docking protein plays a key role in the pathogenesis of atopic dermatitis by inhibiting the activity of CARD11, which leads to the release of interferon IFN-γ by T cells, protecting against the development of atopic dermatitis. DOK3 promotes atopic dermatitis by enabling phosphatase PP4C to inhibit the T-cell-signaling mediator CARD11. Mutations of the CARD11 protein or disturbances of its function are closely associated with the development of severe atopic dermatitis, and its mechanism of action is related to the fact that CARD11 is a critical mediator of antigen receptor signaling in lymphocytes. DOK3, by interacting with CARD11 and reducing its phosphorylation in T cells, suppresses CARD11 signaling, leading to a decrease in the concentration of IFN-γ. Thus, the researchers demonstrated, in an experimental model, that the strength of the interaction of DOK3 with CARD11 may predispose one to the development of atopic dermatitis [14].

The protein induced by this gene turns on signaling pathways involved in the development and functioning cells of the immune system, which leads to abnormalities of T lymphocytes and a weakening of the immune system, and thus to recurrent infections, which are common in people with CARD11-related AD.

It was demonstrated that mast cells and basophils play a major role in atopy, including AD. The important role of Langerhans cells and follicular helper T lymphocytes is also emphasized. Langerhans cells have been shown to direct the follicular helper T cell response when activated directly by thymic stromal lymphopoietin [15].

More than 30 genomic regions that determine the structure and functioning of the epidermis and immunological mechanisms are responsible for atopic dermatitis. One example is a defect in the function of the profilaggrin gene (FLG), occurring in approximately 10% of the population, which causes filaggrin deficiency in the epidermis and weakening of the skin barrier [16].

Another extremely important aspect that guarantees epidermal homeostasis is skin apoptosis, which allows for excess cells to be removed, thus maintaining the proper architecture of the skin and constituting an important anti-cancer defense mechanism in response to UV radiation or oxidative damage.

One of the key proteins conditioning the epidermis and one of the most important factors determining the proper skin barrier is filaggrin, the lack of which disturbs the functions of the diffusion barrier, causing a deficiency of urocanic acid and sensitizing the skin to apoptosis induced by UVB radiation.

Filaggrin binds to keratin fibers in epithelial cells that support our bodies in maintaining a healthy defensive barricade on the skin. The precursor of the amino acids constituting the basic component of the Natural Moisturizing Factor (NMF) is the protein filaggrin. Keratin and filaggrin are the main protein factors of the epidermis (80–90%), especially in its granular layer. The function of filaggrin is to bind keratin fibers together during keratinocyte maturation. As a result of further transformations of filaggrin, compounds that are part of the NMF are formed. In skin diseases involving the abnormal terminal differentiation of epidermal keratinocytes (e.g., atopic dermatitis, ichthyosis, psoriasis), a reduced or complete absence of the NMF is observed [17,18,19]. In addition to the classic T helper type 2 (Th2) cells, type 2 innate lymphoid cells (ILC2), basophils, macrophages and basophils were also shown to be a reasonable source [20]. Therefore, filaggrin is not only a risk factor for AD but also a disease modifier [21].

## 4. Clinical Signs

Skin lesions most often occur on the elbows and knees, face, and neck. Erythema is very clearly demarcated from the surrounding skin, accompanied by erosions, vesicles, and papules. Most often, changes appear on the cheeks, scalp, or forehead. Clinical symptoms depend on whether eczema is acute or chronic. Eczema lesions manifest themselves with erythema, lumps, swelling, scaling, and scratching symptoms, and are not very well defined, while lichenification and xerosis of the elbow extensors are characteristic of chronic eczema. In patients up to 11 years of age, the foci of lichenization are largely localized on the flexion surfaces of the joints. In older patients and adults, changes are also visible on the backs of the hands. There are also cases in which erythroderma can occur on the skin. Moreover, hand eczema is a typical symptom of atopic dermatitis in adults and may also be associated with occupational exposure. Atopic dermatitis is a precursor to the development of food allergies, allergic rhinitis, and asthma. Early-onset acute AD is associated with sensitization to aeroallergens and allergic rhinitis in later childhood.

The disease is diagnosed based on symptoms and signs. Traditionally, the Hanifin and Rajka diagnostic criteria are used, consisting of 4 major and 23 minor criteria. These criteria are the gold standard and should be used in the diagnosis of AD in adults [22]. The major criteria are itching, typical location of lesions, chronic and recurrent course of the disease, and atopy in the patient or other family members. The minor criteria are dry skin beginning in childhood, follicular keratosis and/or ichthyosis, increased IgE concentration in serum, immediate skin reactions, recurrent skin infections, cataract, wool intolerance, food intolerance, exacerbations after mental stress, and white dermographism (vasomotor reaction of the skin). To diagnose AD, it is sufficient to meet three of the four main criteria and three of the minor criteria [23]. Atopic dermatitis may also present with other phenotypes. Eczema, acute or chronic, may be associated with keratosis, ichthyosis, or hyperlinearity, and may have various generalized morphological variants, such as eczema numerical, atopic rash, pityriasis alba, or lichen planus. Additionally, there are localized forms of AD, which include breast dermatitis, retro-auricular intertrigo, periorbital dermatitis, eyelid dermatitis, infra-auricular tears, cheilitis (perlèche), intranasal erosion hand eczema and juvenile palmoplantar dermatitis, and eczema on the tip of the finger (“atopic winter feet”). In turn, on highly pigmented skin, the typical appearance is not red, typical of Caucasians, but gray or ash-gray [24].

Analysis of the various scales used for clinical assessment within the Harmonizing Outcome Measures in Eczema (HOME) initiative resulted in the recognition of the EASI as the recommended primary symptom assessment tool in all clinical trials of AD [25].

The above scale assesses the region (head and neck, upper extremities, trunk, and lower extremities), intensity (0—absent; 1—mild; 2—moderate; 3—sever) and surface area (1 (1–9%), 2 (10–29%), 3 (30–49%), 4 (50–69%), 5 (70–89%), 6 (90–100%)) to provide a total final EASI score that ranges from 0 to 72: a score of 0 indicates no eczema, a score from 0.1 to 1.0 indicates almost clear, a score from 1.1 to 7 indicates mild disease, a score from 7.1 to 21 indicates moderate disease, a score from 21.1 to 50 indicates severe disease, and a score above 51 indicates very severe disease [26].

In turn, the British Working Group proposed diagnostic criteria for AD consisting of one major and five minor criteria. These criteria are a modification of the Hanifin and Rajka scale and include pruritus accompanied by at least three symptoms, including onset under 2 years of age, skin rash, history of asthma or hay fever, dry skin within the past year, and visible flexural dermatitis [27].

Acute eczema is characterized by vesicles and exudative erythema on the cheeks and trunk, mainly in infants. In turn, in childhood and adolescence, lichenization and dryness of the skin, in the folds of the limbs, face, and neck, predominate. The disease can also manifest itself in various phenotypes, but the main subjective symptom is skin itching. Histopathological examination is dominated by lymphocytic infiltration, acanthosis, and parakeratosis, and less often by a psoriatic pattern [28].

The definition of itching (pruritus) describes it as an unpleasant feeling on the skin, causing the desire to scratch. Acute and chronic itching is classified as itching that lasts longer than six weeks. Recent studies have shown that in AD, IL-4, IL-13, and IL-31 directly stimulate sensory neurons responsible for chronic itch, but it is still unclear whether acute itch attacks are the result of the enhanced signaling of already known pathways or whether they use new, different molecular circuits. It was documented that, in AD patients, severe pruritus is associated with allergen-specific IgE [29].

It is known, however, that basophils are responsible for IgE-dependent and mast-cell-independent itch through the released leukotriene C4, which activates sensory nerves, and which also, through neuronal C4-CysLTR2 signaling, is a mediator of acute recurrences of itch. While studies on mice have demonstrated that the mast-cell–histamine axis plays a basic role, under the influence of an allergen, in the occurrence of severe itching, in AD patients, it has been shown that this pathway is not as important as the basophils–leukotrienes–neurons axis, which forms the basis of the neuroimmune process [30].

In summary, mast cells are involved in a variety of neuroimmune processes, but basophils that are rapidly recruited to inflamed tissue are responsible for itching through their pro-inflammatory and neuroimmunomodulatory effects.

## 5. Cytokines in AD

Patients suffering from AD have a disturbed and overactive immune system. The wide clinical spectrum of AD is conditioned by complex mechanisms that lead to inflammation whose severity varies between periods of exacerbation and remission [31,32,33,34,35,36].

By 2010, 37 interleukins had been described and grouped into three families:

1. Interleukin 2 subfamily, which includes IL-3–7, 9, 11–15, 21, 23, 30, colony-stimulating factor (CSF), leukemia inhibitory factor (LIF), prolactin, INF-α, and INF-β.
2. IL-1 subfamily, including IL-1α, IL-1β, and IL-18, along with their derivatives.
3. The third family includes interleukin 17A, B, C, D, F, and IL-8, 25 and 27, as well as IL-31, 32, and 33. The number of described interleukins is still growing: in 2016, 38 interleukins were described, and in 2023, 41 [37,38].

**Figure 1:**
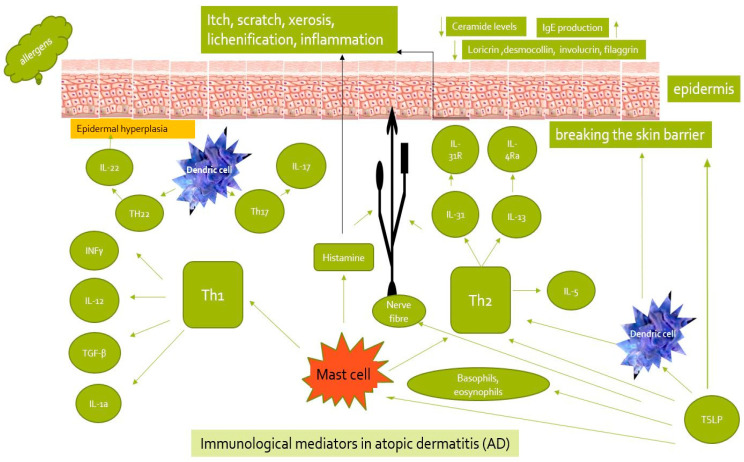
Pathways of immunological mediators in AD.

In AD, the increased production of cytokines such as IL-4, IL-5, IL-10, and IL-13 is observed. Interleukin-1 (IL-1) stimulates the development and maturation of Th2 lymphocytes, while in AD patients, interleukin-4 (IL-4) participates in the regulation of the change in the phenotype of T lymphocytes towards Th2 cells [Figure 1].

Recent reports have shown that IL-13 is a key T2 cytokine causing inflammation in the periphery, while IL-4 has only central effects. This is related to the local overexpression of IL-13, which is responsible for the recruitment of inflammatory cells in the skin, weakening the epidermal barrier and changing the skin microbiome [31].

On the other hand, IL-13 also induces Th2 cell responses and may stimulate Th2 cell development independently of IL-4 [39].

The essential role of interferon γ (IFN-γ) in AD has also been demonstrated by examining the levels of specific chemokines and their receptors on Th1 and Th2 lymphocytes. Patients with AD showed a significantly higher level of the Th1-cell-specific receptor CXCR3 and its ligand Mig (monokine induced by IFN-γ), which is involved in the initiation of Th1 cells. IFN-γ stimulates the expression of the major histocompatibility complex (MHC) of classes I and II, activates monocytes and macrophages, and is involved in the development of Th1 lymphocytes [Figure 1] [40].

Interleukin-13 has a peripheric activity, while IL-4 has a central activity and is responsible for changing the type of immunoglobulins (Ig) produced by B lymphocytes from IgG1, IgG2, IgG3, IgM, and IgE. Interleukin-4 increases the expression of vascular cell adhesion molecule (VCAM), which plays a fundamental role in the migration of eosinophils to the foci of inflammation. Moreover, IL-4 inhibits the response of Th1 lymphocytes, resulting in an increased expression of macrophage inflammatory protein 1 (MIP1) and MIP2, RANTES, and chemokines that re-attract Th1 lymphocytes [41] [Figure 1].

An important cytokine in incurable immune-related diseases is IL-5. The main sources of IL-5 are Th 2 lymphocytes and mast cells. Interleukin-5 mRNA is found in eosinophils from patients with asthma, celiac disease, and eosinophilic heart disease, and IL-5 expression by eosinophils has been confirmed in atopic dermatitis [42].

As research has shown, the inflammatory infiltrate of AD contains eosinophils and the neurotoxins they release, and their amount determines the severity of the disease, hence the concept that IL-5, as the main cytokine influencing the development and functions and prolonging the lifespan of eosinophilia (by inhibiting the apoptosis process), may be the target of biological drugs used in AD.

However, the use of mepolizumab, an anti-IL-5 antibody, in the first clinical trial did not result in a decrease in SCORAD indices and did not significantly affect the pruritus score and the TARC severity marker compared to the control, despite a reduction in eosinophilia [43].

Also, the results of the randomized, double-blind, placebo-controlled, phase II HILLIER study, which assessed the effectiveness of benralizumab treatment in AD, did not show any benefit of using this drug for AD, confirming the complex etiopathogenesis of AD and the fact that eosinophils are only one link in the chain of the complex etiopathogenesis of AD [44].

However, benralizumab, which binds to the IL-5Rα chain and develops its action by initiating antibody-dependent cellular cytotoxicity, has proven to be an extremely effective drug and has already been approved for the treatment of severe forms of eosinophilic asthma. It is involved in both the acute and transient phases, as well as chronic inflammation, stimulating the growth and maturation of eosinophils. By preventing their apoptosis, it increases the release of histamine from basophils, the precursor of eosinophils in the bone marrow [45].

Another important Th2 cytokine is IL-10, which affects the migration of eosinophils, inhibits the cell-type response, and reduces the ability to develop a delayed type reaction and cause contact sensitization. It has been shown that an increased IL-10 concentration and low IFN-γ concentration are the main factors increasing susceptibility to infections in AD patients. Th1 lymphocytes mainly release IFN-γ, IL-2, and IL-12. They play an important role in the cellular response organism during the course of infection, in viral, bacterial, and fungal whiplash, and are involved in the chronic phase of inflammation [41].

Interleukin-12 alters the Th2 response profile to Th1 and is responsible for the transition from the acute to chronic phase by inducing IFN-γ in T cells and inhibiting the production of IL-4 and Th2 cells [46].

Interleukin-18, in the presence of IL-2, induces the secretion of IL-4, IL-10, and IL-13, as well as IFN-γ [47] [Figure 1].

In the early 21st century, several cytokines were characterized, including IL-17B, IL-17C, IL-17D, IL-17E, and IL-17F, which were grouped together and named the IL-17 family. Receptors for the IL-17 family have been discovered, which include IL-17RA and IL-17RE. Interleukin-17 is a pleiotropic cytokine with strong pro-inflammatory properties, the most important role of which is to mediate the pro-inflammatory response by inducing cytokines such as G-CSF, GM-CSF, IL-1β, IL-6, TGFβ, TNF-α, IL-8, GRO-α, MCP-1, and prostaglandins PGE_2_. The pathomechanism of IL-17 action is related to the activation of the nuclear factor NF-κB-dependent signaling pathway, which is dependent on the host TNF-related factor 6 (TRAF6) receptor.

Interleukin-17 has a strong effect on keratinocytes, exerting a chemotactic effect, inducing keratinocyte division and differentiation, inducing the production of chemokines and growth factors, and inhibiting the production of filaggrin, which weakens the dermal–epidermal barrier. Interleukin-17 cytokines play an important role in diseases such as multiple sclerosis, asthma, rheumatoid arthritis, psoriasis and atopic dermatitis, and arthritis; hence, for example, recombinant human monoclonal antibodies that bind IL-17A (secukinumab, ixekizumab, and brodalumab) have been used in the treatment of moderate-to-severe psoriasis. On the other hand, it has been shown that by increasing proliferation and angiogenesis, and activating the formation of metastases, IL-17 plays an important role in the cancer process [48,49,50].

Research suggests a significant role of IFN-γ and/or IL-17A in the “intrinsic” form [51] [Figure 1].

Also, studies conducted in various ethnic groups confirm that Asian patients with AD have different phenotypic features. It has been shown that the incidence of AD in adults is much higher in Asia (7–10%), especially the “extrinsic” form, and correlates with TH17 activation, acute skin lesions with more visible scaling and lichenization, and more clearly demarcated lesions, with higher levels of IgE and eosinophils [52].

Interleukin-23 is a pro-inflammatory interleukin, produced mainly by dendritic cells and activated macrophages, belonging to the IL-12 subfamily consisting of two subunits—p19 and p40—while the receptor for IL-23 and IL-23R, also consists of two subunits, Il23Rα and IL12Rβ1, and is strongly expressed on NK cells, macrophages, dendritic cells, memory T cells, and keratinocytes. Interleukin-23 plays an important role in atopic dermatitis, psoriasis, asthma, inflammatory bowel diseases, rheumatoid arthritis, multiple sclerosis, and viral, bacterial, and fungal infections, as well as in autoimmune thyroiditis and heart attack. Ustekinumab has been shown to inhibit IL-23 by blocking the p40 subunit used in the treatment of psoriasis and psoriatic arthritis and Crohn’s disease [53,54].

In the pathophysiology of allergic diseases, cytokines of epithelial origin, the so-called alarmins (damage-associated molecular patterns (DAMPs)), i.e., TSLP, IL-25, and IL-33, triggered by damaging factors (viruses, allergens, and proteases), are involved in the pathophysiology of asthma and atopic dermatitis by activating the immune response in a Th2-dependent manner [55].

## 6. Atopic Dermatitis Therapy

Current guidelines for the treatment of atopic dermatitis are based on subsequent steps, in which it is recommended to take care of the skin first, i.e., moisturizing, bathing, and avoiding irritating and itching factors. In further management of mild to moderate AD, topical anti-inflammatory drugs, including corticosteroids and topical calcineurin inhibitors, are recommended.

The American Academy of Dermatology recommends first using moisturizers, topical calcineurin inhibitors, and corticosteroids. Baths and compresses are conditionally recommended, but the use of local antiseptics, antihistamines, or antimicrobials is not recommended [56].

Antihistamines are widely used to treat pruritus in AD, but randomized, double-blind studies have not demonstrated the effectiveness of these drugs. Therefore, the American Academy of Dermatology (AAD) guidelines do not recommend the use of non-sedating antihistamines [57].

An analysis of the use of antihistamines in AD, conducted in 1990–1997 by the National American Medical Care Survey (NAMCS), showed that these drugs were prescribed to as many as 20% of patients [58].

Currently, the AAD recommends the use of these drugs mainly in terms of their beneficial effect on insomnia secondary to pruritus; however, this effect is also questioned because there are reports of their negative impact on sleep and cognitive disorders [59].

It is believed that these drugs should be prescribed with caution in patients with comorbid atopic diseases (allergic rhinitis, dermatitis due to food, and conjunctivitis) when relief from troublesome allergic symptoms is expected [60].

In the case of patients with moderate to severe AD, when existing treatment methods are not effective, it is recommended to start advanced systemic therapy, i.e., phototherapy and oral immunosuppressive drugs. Currently, dermatology uses targeted therapies for patients with persistent dermatoses such as psoriasis or atopic dermatitis (Table 1) [61,62]. In the systemic treatment of AD patients, it is possible to use immunosuppressive drugs, biological drugs, and JAK inhibitors (Table 1).

T2 cytokine IL-13 affects transepidermal water loss (TEWL). Also, it was revealed that IL-4 and IL-13 produce AMP through keratinocytes [12,13,14]. Also, IL-13 is involved in the expression of matrix metalloproteinase MMP-9 in human keratinocytes, decreased collagen degradation with subsequent fibrosis, and additional collagen accumulation, which is characteristic for lichenified AD skin lesions. Therefore, pharmacological therapies that inhibit Il-13 biological activity were developed (Table 1) [16,63,64,65].

The results of the phase III clinical trial described by Silverberg et al. are extremely promising, showing the potential use of lebrikizumab in adolescents and adults with moderate to severe atopic dermatitis. After 16 weeks of treatment (283 patients), very good results were contained compared to the control group (141 patients in the placebo group). In study 1, in the study group (283 patients), compared to the control group (141 patients in the placebo group), symptom remission was achieved in 43.1% and 12.7% of patients, respectively, and the EASI-75 response occurred in 58.8% and 16.2%, respectively. In study 2, 33.2% (281 patients) of the lebrikizumab group and 10.8% (146 patients) of the placebo group achieved remission, and EASI-75 response occurred in 52.1% and 18.1%, respectively. Side effects were only mild to moderate [66]. In seriously ill patients, the treatment of choice is biological therapy, which primarily uses anti-IL-4Rα (dupilumab) and anti-IL-13 (tralukinumab) monoclonal antibodies. The undeniable role of Th1, Th17, and Th22 cells has also been demonstrated, the effect of which is the stimulation of Bi cells, the production of IgE, and, through high-affinity immunoglobulin-ε receptors (FcεR1), an effect on eosinophilic cells and the activation of immune reactions via T cells [67]. The role of IL-21 is also considered; high concentrations of this, in addition to its receptors, IL-21R, were found in the epidermis of AD patients, and its concentration correlates with the severity of the disease, assessed using the SCORAD index. Recent studies have shown the presence of IL-21+ T cells surrounded by T cells expressing IL-13 and IL-4R in the skin of AD patients, which confirms the effectiveness of AD treatment with JAK1 inhibitors such as abrocitinib, baricitinib, and updacitinib [68,69]. Further observations and studies indicate a large role of IL-18 in the assessment of AD activity, while IL-18 inhibitors may be a potential therapeutic target for AD [66].

Dupilumab is a monoclonal antibody, injected subcutaneously, that binds to the alpha subunit of the IL-4 receptor, blocking the IL-4 and IL-13 signaling pathways. The drug is approved for children over 6 years of age. The conducted studies have shown that, after 3 months of treatment, an almost 75% improvement in the clinical condition is achieved in approximately 50% of patients when the drug is used as a monotherapy and in 70% of patients in the case of combined treatment with the local application of glucocorticosteroids. The side effects are minor: only mild conjunctivitis and local reactions at the injection site are reported. Another antibody that binds to IL-13 is Tralokinumab, approved for the treatment of moderate to severe atopic dermatitis from the age of 12 years. Phase III studies of the drug used for 16 weeks showed an improvement in the clinical condition in over 75% of patients, including approximately 30% of patients after monotherapy and 56% of patients after combination therapy with a class 3 local glucocorticosteroid [64,70,71].

Dupilumab is believed to reverse the epidermal barrier defect by beneficially interfering with the molecular and cellular mechanisms underlying AD [65].

Chu et al. [72] reviewed 149 clinical trials that enrolled 28,686 patients with moderate to severe AD and analyzed 75 therapeutic options. Based on this, the authors concluded that high-dose upadacitinib is one of the most effective drugs, followed by high-dose abrocitinib and low-dose upadacitinib. Unfortunately, these Janus kinase inhibitors have caused many side effects. Dupilumab, lebrikizumab, and tralokinumab demonstrated intermediate efficacy and mild side effects (mild conjunctivitis). Among the biological drugs, baricitinib was the least effective, as were the immunosuppressive drugs—azathioprine, oral corticosteroids, cyclosporine, methotrexate, and mycophenolate—which were associated with additional consequences and numerous side effects [16,65,72].

In November 2023, the European Commission (EC) approved the drug EBGLYSS (lebrikizumab) for the treatment of patients with moderate to severe AD in both adults and adolescents (aged 12 years and older) weighing at least 40 kg.

Lebrikizumab is a monoclonal antibody that binds to IL-13 to block the formation of the IL-13Rα1/IL-4Rα heterodimer complex and inhibit the action of IL-13, which plays a key role in AD. It seems that this biological drug may become a first-line drug, setting a new paradigm for the treatment of AD [Table 1]. The European Commission’s approval was the result of the results achieved in three pivotal phase III studies: ADvocate 1 and ADvocate 2 (lebrikizumab monotherapy) and ADhere (lebrikizumab in combination with topical corticosteroids (TCS). Lebrikizumab was characterized by early clinical effectiveness already in the 16th week of treatment, reducing the severity of the disease by over 75% (EASI-75) in 6 out of 10 patients and in 7 out of 10 patients receiving combination therapy with a corticosteroid, without observing any serious side effects (reactions in injection site, conjunctival dryness and effects on the eye) [73].

Other biological drugs directed against the IL-13Rα1 receptor in clinical trials include ASLAN004 and Anrukinzumab (IMA-638), while RPC4046 and CNTO 582599 block the binding of IL-13 to both IL-13Rα1 and IL-13Rα2. It was revealed that there are a few possible pathways for targeting IL-13 [73,74].

The role of tralokinumab is to bind to the IL-13 cytokine. These processes are located at an epitope that overlaps the IL-13Rα receptors [44]. The main process in AD immunopathogenesis is changing the regulation of helper T-cell type 2 (TH2) cells and type 2 innate lymphoid cells. During this process, an increase in type 2 immune cytokines is noted, especially IL-4, IL-13, and IL-31. Recently, IL-37, IL-26, the IL-17 and IL-31/33 axis, and thymic stromal lymphopoietin (TSLP) were demonstrated to play a significant role in the immunopathogenesis of AD [Figure 1] [75].

**Table 1:** Characterization of cytokines in terms of their role in AD.

Cytokine	Mechanism of Action	Treatment	Reference
IL-2	Atopic dermatitis is associated with decreased lymphoproliferative responses upon stimulation with T-cell mitogens, depending on IL-2, which, in turn, upregulates the expression of its own receptor.	Cyclosporine	[76]
IL-4	Type 2 immunity is considered central to AD pathogenesis and key therapeutic targets.	Dupilumab—Phase III,	[77]
IL-12/23	Th1 response and influence on the IFN-y release [8],	Ustekinumab—Phase II Study [20]	[78,79]
IL-13	Blocking interleukin-13 with targeted therapies; activation of TRPA1.	Lebrikizumab—Phase I and Phase III. Tralokinumab—Phase II	[77]
IL-17	Secreted by Th17. IL-17A-IL-17F cytokines possess proper receptors. IL-17C is a unique cytokine.	Ustekinumab—Phase II Study Secukinumab phase II trial is a monoclonal antibody against IL-17A for the treatment of psoriasis, psoriatic arthritis, and ankylosing spondylitis. MOR106 p/monoclonal antibodies against IL-17C	[79,80]
TLSP	TSLP increased compared to healthy people.	AMG157—Phase I trial Tezepelumab—Phase II MK8226—Phase I	[76,81]
IL-22	IL-22, with IL-17, triggers antimicrobial peptide; part of the IL-10.	ILV-094—Phase II, ongoing	[82,83]
IL-31	IL-31 receptor [IL-31R].	Nemolizumab—Phase II, phase III	[84,85]
IL-24	Type 2 cytokines; has a high level in AD patients; is responsible for inflammation, itch and hyperplasia in AD.		[86]
IL-18	Il-18 stimulates release of IL-4, IL-5, IL-9, and IL-13.		[78]
IL-37	IL-37 binds to IL-18R.		[87]
IL-19	IL-19 is significantly elevated and correlated with EASI scores, and decreases with skin treatment.		[88,89]
IL-26	Increased concentration of IL-26 activates keratinocytes to release cytokine and stimulates T.		[90]
IL-33	IL 33 has an affinity for a Toll receptor (TLR)/IL1R), which forms a heterodimer with the IL-1-receptor-associated protein (IL-1RAcP) and stimulates the immune cascade.	1. Etokimab in (NCT02920021) clinical trials 2. Etokimab phase 2a clinical trial for adults with eosinophilic asthma (NCT03469934). 3. Etokimab phase 2b clinical trial for adults with moderate-to-severe atopic dermatitis 4. Etokimab in phase 2 clinical trial	[91,92]
OX40	The important role of the T cell co-stimulatory tumor necrosis factor receptor (TNFR) OX40 and its cognate ligand, OX40L, in autoimmune diseases, including AD.	GBR 830—human monoclonal IgG1 antibody specific to OX40 (CD 134). GBR 830 was assessed in a phase Iia, randomized, double-blind, and placebo-controlled study in AD patients	[93]

IL-4 stimulates the production of increased levels of IgE. Interleukin-4 and IL-13 genes are located in a region of 140 kb on chromosome 5q31-33, which codes for a cluster of Th2 cytokines, and gene polymorphisms play an important role in the pathogenesis of atopic diseases, including AD, but also allergic rhinitis, idiopathic nephrotic syndrome, and asthma [94].

Interleukin-4 interacts via IL-4Rα, expressed on B and T cells, macrophages, and others. IL-4 and IL-13 are obligatory factors promoting Th2 cell differentiation and class switching into IgE in B cells [95,96,97].

One way to treat AD is with Lebrikizumab. Lebrikizumab inhibits IL-13Rα1/IL-4Rα. The method of treatment with lebrikizumab is dose-dependent, and the effectiveness of the treatment varies [98]. Another biological treatment option is tralokinumab. A Phase Iib, randomized, multi-dose study (NCT02347176) was conducted to investigate the efficacy, safety, and tolerability of tralokinumab in adults diagnosed with AD. The most common adverse event was upper respiratory tract infection, which was reported to be related to the placebo (3.9%) and combined tralokinumab (3.9%) groups. The drug was well tolerated by patients, and when administered early, it allowed for lasting improvement of the symptoms of the disease in people with moderate and severe forms of AD, but the authors emphasize that phase III studies are needed to confirm these results [73,99,100,101,102].

Ustekinumab is another drug used to treat AD. Ustekinumab is a human IgG1κ monoclonal antibody that binds with high specificity to the p40 protein subunit common to IL-12 and IL-23 cytokines, inhibiting their activity and preventing the binding of these cytokines to their IL-12Rβ1 protein receptors located on the surface of immunocompetent cells. It is an IgG1 monoclonal antibody against p40 [103,104].

Husein-ElAhmed H et al. [105] reviewed the literature on the use of ustekinumab in the treatment of 23 AD patients, assessing its effectiveness in three categories—‘complete response’, ‘partial response’, and ‘no response’—showing complete remission in eight patients (34.8%), partial remission in seven (30.4%), and no response in eight patients (34.8%). This gave rise to the authors’ conclusion that the use of ustekinumab to block the IL-12/23p40 pathway is not as effective in the treatment of AD as, for example, the use of antibodies targeting IL-4, I-13, or I-31.

Interleukin 22 promotes epidermal hyperplasia and inhibits skin barrier function. IL-22 also increases antimicrobial activity and S100A7, S100A8, and S100A9 in keratinocytes [106,107,108,109,110,111,112,113].

The use of secukinumab against IL-17A is registered as a treatment of psoriasis, psoriatic arthritis, and ankylosing spondylitis [95]. A II trial, randomized, double-blind trial with secukinumab was performed on 44 (22 patients with an intrinsic and 22 with an extrinsic form) [112]. Another antibody effect against IL-17C MOR106 in the RDBPC clinical trial phase was evaluated in 25 patients with AD [113]. Interleukin-19 is a pro-inflammatory cytokine that has its place in the IL-10 family of cytokines (like IL-20, 22, 24, and 26). Interleukin-19, influenced by IL-17A, is powerfully expressed in skin lesions of AD patients. Thymic stromal lymphopoietin (TSLP) is an epithelial-derived cytokine similar to IL-7, and its action manifests itself through the TSLP-Receptor (TSLP-R). TSLP is a strong barrier against risk signals in the skin and lung. It was shown that high levels of TSLP play a key role in atopic inflammation and are characteristic of the patients, children, and adults who suffer from AD [89,114,115,116]. TSLP is highly expressed in the epidermis of patients with AD, and its production is activated through exposure to environmental factors, such as cigarette smoke, allergens, chemicals, microorganisms, and diesel exhaust [117].

## 7. Anti-JAK Therapy

The mechanism of action of Janus kinase inhibitors (JAKi, JAK inhibitors, and jakinibs) involves inhibiting the activity of enzymes from the Janus kinase family (JAK1, JAK2, JAK3, and TYK2), interfering with the JAK-STAT signaling pathway.

JAK and STAT proteins bind to type I/II cytokine receptors, thereby becoming activated and phosphorylating STAT proteins, which move to the nucleus to activate gene transcription. There are four members of the JAK family (JAK1, JAK2, JAK3, and TYK2) and seven members of the STAT family, which actively bind to cytokine receptors responsible for inflammation and itching. These cytokines include IL-4, IL-5, IL-13, IL-31, IL-22, and TSLP [118].

In recent years, oral and topical Janus kinase (JAK) inhibitors have been introduced into the treatment of atopic dermatitis. The advantage of using topical JAK inhibitors is the direct application of the drug to skin lesions and the reduction in the risk of serious side effects observed after treatment with oral drugs, including skin infections and cardiovascular events (MACE), thromboembolic events, and cancer.

The first drug from this group to be registered for the treatment of atopic dermatitis was ruxolitinib (RUX) [119].

In an 8-week, phase II study, RUX cream was used in patients over 12 years of age with atopic dermatitis and showed excellent anti-inflammatory and antipruritic effectiveness without any significant complications (Table 1) [120].

Another JAK inhibitor that is used topically was Delgocitinib. Studies of this drug were conducted in Japan as a randomized, double-blind, 28-week, vehicle-controlled study, and an open-label, long-term extension study was conducted of adult patients with moderate to severe atopic dermatitis. The study’s conclusions described the drug as effective and free of serious side effects [121].

Among the oral JAK inhibitors, we can distinguish abrocitinib, baricitinib, and upadacitinib.

Analyses of the effectiveness of the above drugs showed that abrocitinib, baricitinib, and upadacitinib are an effective form of therapy in adults and adolescents with AD, as shown in a comparative efficacy and safety study for moderate-to-severe atopic dermatitis using a network meta-analysis [122].

The phase IIb study of abrocitinib included 267 adult patients with moderate to severe atopic dermatitis, showing the high effectiveness of the drug, but reporting side effects, among which the most serious were pneumonia, pulmonary embolism, thrombocytopenia, nausea, headaches, and eczema herpeticum. These symptoms disappeared after 4 weeks of continued treatment at the latest. Recently, the results of the phase III JADE MONO-1 and JADE MONO-2 studies were published, which included 387 patients with AD. TEAEs were reported in 65.8% (200 mg abrocitinib), 62.7% (100 mg abrocitinib), and 53.8% (placebo) of patients, respectively. Side effects included nausea, headache, nasopharyngitis and upper respiratory tract inflammation, thrombocytopenia, and hyperlipidemia. It was concluded, based on the phase II and III studies, that abrocitinib may be a safe alternative to conventional AD therapies [123,124].

Baricitinib also demonstrated a high safety and effectiveness profile in 124 adult patients with AD in a phase II clinical trial. TEAEu of 49% (placebo), 46% (baricitinib 2 mg), and 71% (baricitinib 4 mg) were reported. Phase III BREEZE-AD1 and BREEZE-AD2 studies did not show any serious complications, except for headaches and inflammation of the upper respiratory tract. The use of emerging systemic JAK inhibitors in the treatment of atopic dermatitis was reviewed [125].

The phase III clinical trial with upadacitinib, which included 901 patients that received upadacitinib 15 mg and topical corticosteroids (*n* = 300), upadacitinib 30 mg plus topical corticosteroids (*n* = 297), or a placebo with corticosteroids topically applied (*n* = 304), indicated that upadacitinib in combination with topical corticosteroids was more effective and well tolerated compared to the placebo [126].

## 8. Anti-TSLP Therapy, Anti-OX40

Tezepelumab is an antibody directed against circulating TSLP, which was evaluated in a three-part study [91]. Interleukin-31 is a cytokine belonging to the IL-6 family of cytokines. Upon activation, as well as by CD45RO + T cells, IL-31 mRNA is expressed by CD4 + T helper 2 (TH2) cells, which are associated with the skin antigen associated with the skin lymphocyte. In addition, basophils, eosinophils, and mast cells secrete IL-31 and have the potential to be associated with chronic pruritus. Interleukin-31 acts through the IL-31 A receptor (IL-31RA) and the Oncostatin M receptor (OSMR). The contribution of IL-31R via IL-31 triggers the JAK1/JAK2 and STAT3 and the ERK1/2, p38 MAPK, PI3K/Akt, and JNK pathways. There are two forms of IL-31RA (short and long), while both activate JAK1/JAK2 and STAT3 signaling in an OSMR-dependent manner. Several new drugs are currently being assessed in clinical trials and have obtained drug approval, including dupilumab (an anti-IL-4-α receptor antibody), 49 phosphodiesterase inhibitor 4.50.51 (crisaborole), and a JAK inhibitor [127]. The antipruritic effect of these new anti-inflammatory drugs may be attributed, in part, to a decline in IL-31 signaling [128].

Epidermal keratinocytes and the sensory nerve fibers of AD patients express high levels of IL-31RA in comparison to healthy ones. A novel therapeutic strategy for targeting pruritus and inflammation, and restoring of the damaged skin barrier associated with AD, is the use of Nemolizumab, an anti–IL-31 receptor humanized monoclonal antibody that was developed for targeting IL-31RA receptors, inhibiting binding between IL-31 and IL-31RA [129].

A double-blind, randomized, placebo-controlled, phase I/Iib clinical trial using nemolizumab was performed in humans and revealed that the anti–IL-31 antibody improves the clinical symptoms and decreases pruritus associated with AD [130,131].

Kwatra et al. presented the results of a phase III clinical trial and revealed that nemolizumab monotherapy significantly reduced the signs and symptoms of prurigo nodularis in 183 patients assigned to the treated nemolizumab group from 274 patients after randomization (91 patients in placebo group) [132].

Another cytokine contributing to the occurrence of AD and produced by Th17 cells is IL-26 [34,133]. Interleukin-26 expression is regulated by IL-1β, IL-23, and RORγt, and its main sources are NK, Th1, and Th17 cells. This cytokine is unique because it has dual functions. Firstly, it has a pro-inflammatory effect by stimulating the secretion of pro-inflammatory cytokines and chemokines by myeloid cells and inducing Th17 cells. Secondly, it has antimicrobial (bactericidal and antiviral) effects. Morita et al. revealed that patients with AD have a higher level of IL-2 in the blood and IL-2 production by T-cells is higher in this group of patients. Treatment using Tranilast (N-3, 4-dimethoxycinnamoyl anthranilic acid) provides a decrease in the IL-2 production of T cells in AD patients [134]. Tranilast, thanks to its anti-allergic properties, was approved in Japan and South Korea in 1982 for the treatment of bronchial asthma, keloid, and hypertrophic scar, but currently has no approval in the United States or elsewhere. Tranisilab works by inhibiting the release of histamine from mast cells, inhibiting fibroblast proliferation and the release of multiple cytokines in various cell types. A clinical trial (PRESTO) also assessed the effect of this drug in preventing restenosis after percutaneous coronary revascularization, but its effectiveness in this indication was not demonstrated [135].

Salamon et al. confirmed that IL-33, in association with IgE, is perilous to MC-IL-2 production. The authors showed that the high content of IL-33 and IgE, along with its infiltration in the dermis, stimulate the production of IL-2 by MC, which activates Tregs and indicates a complex mechanism of atopic dermatitis, which includes the anti-inflammatory effect of MC, IL-33, and IgE, which are conventionally considered pro-inflammatory factors [136].

### 8.1. IL-18

Studies have shown that the IL-18 rs187238 polymorphism affects the risk of AD in the general population. Interleukin-18 in the presence of IL-2 induces the secretion of IL-4, IL-10, and IL-13, as well as IFN-γ, and is a participant of the IL-1 family that increases both inborn and acquired immune response [137]. Interleukin-18 is involved in both Th1-and Th2-type diseases, produced from T cells, B cells, keratocytes, NK cells, macrophages, dendritic cells (DC), and chondrocytes, and stimulates the production of Th 2 cytokines and IgE, together with IL-12. Zedan et al. conducted a serum analysis, showing higher levels of IgE, IL-18, or IL-12/p40 in AD patients compared with the controls [138]. The obtained data visibly demonstrate that IgE, IL-18, and IL-12 could be useful in estimating atopic dermatitis activity, and could be helpful in forecasting the advance of the disease.

Among the five different IL-37 join variants, IL-37b is the most operative variant [102]. Hou et al. suggested that IL-37b could be a potential anti-inflammatory cytokine for AD treatment due to its influence, which significantly upgrades eosinophils-mediated allergic inflammation via the regulation of the autophagy mechanism, the intestinal bacterial assortment, and their metabolites in AD [139].

Interleukin-33 mRNA and proteins are significantly increased in the inflamed skin lesions of AD patients when compared with non-inflamed skin. The localization and accurate mechanism of IL-33 have not been completely explained. Stimulation of Th2-polarized CD4 + T cells by IL-33, releases IL-4, IL-5 and IL-13 (from mast cells and basophils). Imai reported that IL-33 effectively induced the rapid expansion of ILC2s, and this pathway releases many cytokines, especially IL-5 and IL-13 [140]. Based on the results of the Imai studies, it seems that the mechanism of IL-33 is to induce innate inflammation in AD.

P. Salamon et al. performed a preclinical evaluation of a human IgG4PAA mAb with high affinity and specificity for IL-33, torudokimab, which became the subject of a clinical trial using this drug in atopic dermatitis [136].

Okragly et al. showed the complete neutralization of IL-33 activity in vitro and in vivo in cynomolgus monkeys; hence, great hopes are associated with this drug and its use to treat atopic dermatitis in clinical trials [141].

This resulted in phase II and III clinical trials, which allowed for the registration of this drug for the treatment of asthma and allergic dermatitis. The studies were conducted in 9 countries, in 58 centers in Asia, Europe, South America, and North America, with patients with moderate to severe atopic dermatitis who were administered subcutaneous injections every 4 weeks for 16 weeks. However, the study was discontinued due to the lack of effectiveness of the preparation. Of the 136 patients, 48 (35.3%) completed 16 weeks of treatment, while 32 (23.5%) discontinued due to study withdrawal, adverse events, lack of efficacy, or pregnancy. Fifty-six people (41.2%) discontinued treatment. The latest studies confirm the expression of IL-33 receptors in atopic dermatitis, but indicate that IL-18 may be of key importance in maintaining the independence of IL-33 activation of ILC2 during skin inflammation, hence the low effectiveness of IL-33 inhibition in atopic dermatitis [142].

It was also reported that IL-33 induces IL-31 and promotes pruritus. A positive association was reported between the serum levels of IL-31 and the severity of AD [89,113,137,138]. Similarly, treatments directing IL-33 may be accepted in the future. Interleukin-33 can enhance the lipopolysaccharide-mediated in vitro initiation of macrophages, with the upregulation of the expression of toll-like receptor (TLR)4, myeloid differentiation protein 2, soluble CD14, and MyD88 [114]. A Phase 2a, placebo-controlled, 6-week study of etokimab was performed to assess the efficiency and safety of this anti-IL-33 antibody in peanut-allergic adults (NCT02920021). Researchers revealed increases in the tolerated threshold allergen dose at days 15 and 45 of a food challenge (accepting a cumulative 275 mg of peanut protein), changing from 73% vs. 0% to 57% vs. 0%, respectively. The authors concluded that the phase 2a results demonstrate etokimab as an effective, safe, and well-tolerated treatment in peanut-allergic participants [143].

### 8.2. Etokimab

Interleukin-33 is an early inflammatory mediator, acting as an alarmin secreted by epithelial cells in the event of cellular stress and damage, signaling danger and mobilizing the immune system to respond. Recent studies have shown that it is important in AD and asthma. Tests of the new therapy were carried out on 12 adult patients with moderate and severe atopic dermatitis, showing that giving patients a single dose of the drug significantly improves their clinical parameters, skin condition, and quality of life even 20 weeks after administration. Etokimab was also used in clinical trials to assess the efficacy of anti-IL-33 therapy in atopic dermatitis. Patients who obtained etokimab showed a 40% decrease in SCORAD at day 29. Etokimab was well-tolerated by all patients [144,145,146].

### 8.3. OX40

The expression profile of OX40 has been recognized as being primarily activated on CD4+ T cells [112]. GBR 830 expressively reduced the mRNA expression of immune modulators Th1 axis—IFN-y, CXCL10, Th3 axis—IL-31, CCL11, CCL17, and TSPLR and Th17/Th22 axis—IL-23p19, IL-8, S100A9, and S100A12 by day 71 [144]. In GBR 830, post-treatment lesion skin reductions in OX40+ T cells and OX40L  +  dendritic cells were also noted.

E. Guttman-Yassky published promising research results in Lancet on the use of Rokatinlimab, which inhibits the OX40 molecule in patients with moderate and severe AD. The Phase 2b, multicenter, double-blind, placebo-controlled study evaluated 274 patients at 65 sites in the United States, Canada, Japan, and Germany (rocatinlimab: *n* = 217; placebo: *n* = 57). Rocatinlimab was randomly administered intramuscularly every four weeks (150 mg or 600 mg) or every two weeks (300 mg or 600 mg), or a placebo was administered subcutaneously until week 18, with an 18-week extension of active treatment and 20-week follow-up. The percentage eczema area size and severity (EASI) compared to baseline was assessed as the primary endpoint at week 16, and significance was achieved for all active doses of rocatinlimab (from −48% to −61%) compared with placebo (−15%). All treatment groups improved after week 16, and the response lasted for at least 20 weeks after treatment. Thus, it was confirmed that rocatinlimab is a safe and effective drug for moderate and severe AD, without any serious side effects, apart from fever, chills, headaches, canker sores, and nausea [147].

### 8.4. IL-24

CD4+ T cells, NK cells, keratinocytes, bronchial epithelial cells, mast cells, and myofibroblasts produced cytokines. It was confirmed in many research studies that IL-24 plays an important role in diseases such as psoriasis, arthritis, and or inflammatory bowel diseases. It has been revealed that IL-24, IL-20 IL-19, and IL-22 are dysregulated in injured skin and have crucial roles in barrier dysfunction and S. aureus infection [86]. Cornelissen et al. described that IL-24 and IL-20, generated in IL-31–stimulated keratinocytes, decreased the expression of filaggrin [148]. Bożek et al., in 38 children with co-occurring AD and PS, 41 patients with AD only, and 28 patients with PS only, performed a clinical assessment using the SCORAD and PASI scales and found the following cytokines in the serum using the ELISA test: IL-2, IL-4, IL-5, IL- 6, IL-8, IL-12, IL-17, IL-18, IL-22, IL-33, TNF-α, IFN-γ, and TARC/CCL17. The author showed a high level of IL-17 in patients with coexisting AD and PS, even higher than that found in patients with only one of the diseases. Moreover, he showed that the concentration of IL-22 in serum was significantly higher in patients suffering only from AD compared to other patients [149].

In another study, Krupka-Olek et al. assessed 39 adult patients with comorbid psoriasis (PS) and AD (ADPS), determining the cytokine profile and comparing the results with 45 people with homogeneous PS or 47 with AD, and 42 healthy people, constituting the control group. Clinical assessment was made using the SCORAD and Psoriasis Area Severity Index (PASI) indices and immunological assessment by determining the cytokines IL-2, IL-4, IL-5, IL-6, IL-8, IL-12, IL-17A, IL-18, IL-22, IL-33, TNF-α, and IFN-γ. Researchers noted significant differences in IL-17A concentrations between patients with ADPS (16.1 ± 5.4 pg/mL), AD (5.8 ± 2.1 pg/mL), or PS (5.0 ± 3.1 pg/mL) and the control group (3.3 ± 1.8 pg/mL), thus showing the important role of T17 cells in the pathogenesis of ADPS [150].

## 9. Conclusions

The pathogenesis of AD is complex and not fully understood. The results of clinical studies confirm the basic role of immunological and non-immunological factors, including the key role of Langerhans cells, dendritic cells, IgE immunoglobulins, and lymphocytes. However, in recent years, there has been a strong development of modern methods of treating AD using two main groups of medicinal products: monoclonal antibodies that act accurately to inhibit specific cytokines or their receptors, and agonists or antagonists of small molecules (abrocitinib, baricitinib, upadacitinib, and tofacitinib). The differences between monoclonal antibodies and small molecular inhibitors are as follows: production mechanisms biotechnology vs. chemical synthesis), route of administration (subcutaneous/intravenous vs. oral/topical), and target (extracellular surface or soluble molecules vs. intracellular molecules). A data analysis indicates that Th2 cells and their associated cytokines, such as IL-4, IL-13, and IL-31, play an essential role in AD. Based on the current literature, it can be concluded that one of the most effective methods of treating atopic dermatitis is the inhibition of IL-4/IL-13 signaling, which makes it possible to restore the optimal skin lipid composition and improve the skin barrier function in patients with moderate to severe atopic dermatitis. Therefore, many new strategies are currently being developed, including targeting this cytokine and its receptors. Biologics are currently at a key stage in their clinical development programs. Interleukin-4 interacts via IL-4Rα, expressed on B and T cells, macrophages, and others. Interleukin-4Rα provides differentiation to Th2 and IgE class converting in B-cells and mast cells, which is responsible for inflammation. The targeted IL-13 inhibitors (tralokinumab, lebrikizumab, cendakimab, and eblasakimab) have been approved for the treatment of moderate to severe atopic dermatitis, offering treatment efficacy, safety, persistence over time, and a significant improvement in pruritus and quality of life. Understanding the pathophysiology and triggers of AD is crucial for effective management and treatment, as AD significantly impacts the quality of life of those affected by this skin disorder. The results of current clinical trials give us great hope for the possibility of introducing monoclonal antibodies that inhibit specific cytokines or their receptors and agonists or antagonists of small molecules into the AD treatment armamentarium. The effectiveness and safety of such treatments in different forms of AD has already been confirmed, and the coming years will allow for the implementation of this modern treatment regimen for AD [151]. Recent genetic studies (GWAS) provide hope that we can expand our knowledge of AD. Since the publication of the EARly Life cycle genetics and epidemiology (EAGLE GWAS) meta-analysis in 2015 by Buddu-Agrey et al., 81 loci were identified among people of European descent, reproduced in a subsequent sample of 2.9 million Europeans individuals, as well as 29 new associated loci and 10 additional loci in multiple-ancestry analyses (three novel). Demographic differences between Europe, Africa, East Asia, and Latin America have also been elucidated [152]. Eight variants were replicated in at least one tested population (European, Latin, or African), but two are specific to individuals of Japanese origin. The authors showed that AD loci showed increased DNase I sensitivity and eQTL associations in the blood. In their findings, the authors emphasize that these genes are mainly involved in immune pathways relevant to atopic inflammation, and some of them may provide new perspectives on the optimal treatment of AD [152]. The above research opens new perspectives and possibilities for the treatment of atopic dermatitis.

## Data Availability

All data have been included.
